# The Gs-Linked Receptor GPR3 Inhibits the Proliferation of Cerebellar Granule Cells during Postnatal Development

**DOI:** 10.1371/journal.pone.0005922

**Published:** 2009-06-15

**Authors:** Shigeru Tanaka, Imran Mohammed Shaikh, E. Antonio Chiocca, Yoshinaga Saeki

**Affiliations:** Dardinger Laboratory for Neuro-oncology and Neurosciences, Department of Neurological Surgery, The Ohio State University, Columbus, Ohio, United States of America; Medical College of Georgia, United States of America

## Abstract

**Background:**

During postnatal murine and rodent cerebellar development, cerebellar granule precursors (CGP) gradually stop proliferating as they differentiate after migration to the internal granule layer (IGL). Molecular events that govern this program remain to be fully elucidated. GPR3 belongs to a family of Gs-linked receptors that activate cyclic AMP and are abundantly expressed in the adult brain.

**Methodology/Principal Findings:**

To investigate the role of this orphan receptor in CGP differentiation, we determined that exogenous *GPR3* expression in rat cerebellar granule neurons partially antagonized the proliferative effect of Sonic hedgehog (Shh), while endogenous *GPR3* inhibition by siRNA stimulated Shh-induced CGP proliferation. In addition, exogenous *GPR3* expression in CGPs correlated with increased *p27/kip* expression, while *GPR3* knock-down led to a decrease in *p27/kip* expression. In wild-type mice, *GPR3* expression increased postnatally and its expression was concentrated in the internal granular layer (IGL). In *GPR3* −/− mice, the IGL was widened with increased proliferation of CGPs, as measured by bromodeoxyuridine incorporation. Cell cycle kinetics of *GPR3*-transfected medulloblastoma cells revealed a G0/G1 block, consistent with cell cycle exit.

**Conclusions/Significance:**

These results thus indicate that GPR3 is a novel antiproliferative mediator of CGPs in the postnatal development of murine cerebellum.

## Introduction

In the adult, the cerebellum is organized into distinct layers, each containing specialized neuronal cell populations, positioned there during postnatal development with precise coordination of processes involving cell proliferation, differentiation, and migration. In the immediate postnatal period, the outermost layer, the external granule layer (EGL), contains dividing granule neuron progenitors (GNP). These granule cells migrate inwards, guided by Bergmann radial glial fibers through the molecular layer (ML), pass through the Purkinje neuron layer (PL) containing the cell bodies of Purkinje neurons and Bergmann glia and finally settle into the internal granular layer (IGL) where they exit the cell cycle and terminally differentiate[1]–[8]. This process is relatively rapidly: in fact, by the end of the third postnatal week in the mouse, the EGL has ceased to exist and the shape of the cerebellum has been transformed from its embryonic flat, oval form to a more complex structure with deep fissures and large folia.

The molecular events responsible for this “switch” from proliferation in the EGL to cell cycle exit and differentiation in the IGL over the 3 weeks of postnatal development are only partially understood. The Hedgehog (HH) signaling pathway, an important mitogenic signal for GNPs[9], is the likely factor that regulates granule progenitor cell (GPC) proliferation [10]–[12], [13]. Indeed, mutations that activate the Hedgehog pathway contribute to the formation of medulloblastoma [14]–[20], [21]. Sonic hedgehog (Shh) is the most widely studied member of this pathway [22]–[25], [26], and one of its most important targets is to activate the Gli family of transcription factors, including transcription of the Gli family of transcription factors [27], [28]. Shh responses can be antagonized by increasing cAMP levels and PKA activity [29], [30], [31]. Regulators of Shh signaling include pituitary adenylate-cyclase activating peptide (PACAP) that raises PKA activity and antagonizes Shh [31], [32], [33]. p27/Kip1 is another factor that has been reported to be predominantly expressed in the developing cerebellum and to play a critical role in the regulation of GCP proliferation [34] by promoting cell cycle exit of GCPs and antagonizing Shh action.

The G-protein coupled receptor GPR3 (GPCR21) is a constitutive activator of intracellular cAMP [35]–[37]. GPR3 belongs to a family of G protein coupled receptors (GPCR), predominantly expressed in mammalian brain. Up-regulation of these receptors in cultured cells stimulates adenylate cyclases to levels similar to those obtained with other G_s_-coupled receptors that have been fully stimulated by their ligands [36], [37]. GPR3 is expressed in oocytes and maintains meiotic arrest with a Gs-dependent mechanism [38], [39].

We hypothesized that GPR3 activation may represent one of the signals that mediates the postnatal cell cycle exit and terminal differentiation of GPCs. This hypothesis was based on the finding that expression of *GPR3* up-regulates intraneuronal cAMP [40]. In addition, *GPR3* mRNA expression was strongly increased in the IGL during cerebellum development [40]. In this report, we show that *in vitro GPR3* expression antagonizes Shh and is associated with p27/kip expression. *In vivo*, absence of *GPR3* leads to a hyperproliferative response of CGPs in IGL, suggesting that it may be the granule cell receptor that mediates cAMP's action on cell cycle exit of CGPs during postnatal development.

## Materials and Methods

### Ethics statement/Animals

All animal experiments were performed according to the guidelines of the Subcommittee on Research Animal Care of The Ohio State University, a IACUUC approved veterinary facility. C57BL/6 mice were obtained from Charles River Laboratories (Wilmington, MA) and SD rats were from Harlan Laboratories (Indianapolis, IN). The *GPR3−/−* knockout mice were obtained from Deltagen (Redwood City, CA).

### Chemicals, proteins, and siRNAs

Recombinant mouse Shh C-terminal peptide was purchased from R&D System (Minneapolis, MN). db-cAMP were purchased from Calbiochem (La Jolla, CA). All other chemicals were from Sigma (St.Louis, MO), unless indicated otherwise. G Silencer® siRNA against rat *GPR3* (5′-CCUACUACUCAGAGACAACtt/5′ GUUGUCUCUGAGUAGUAGGtg) and negative control #1 siRNA were purchased from Ambion, Inc. (Austin, TX).

#### Isolation of rat cerebellar granule neurons and DNA electroporation

Cerebellar granule neurons were isolated from Sprague Dawley rats (Harlan, Indianapolis, IN) according to a published procedure [6], [41] with modifications. Briefly, pups were sacrificed at postnatal day 7 (P7) and the whole cerebellum was removed. The meninges were carefully stripped off, and whole tissue was then washed in calcium- and magnesium-free phosphate-buffered saline (PBS: 10 mM sodium phosphate, 0.9% sodium chloride pH = 7.3) and dissociated into single cells using the Worthington Papain Dissociation System (Worthington Biochemical Corporation, Lakewood, NJ). The dissociated cerebellar cells were applied immediately onto a two-step gradient of Percoll (35%/60% in PBS; Sigma-Aldrich). After centrifugation at 2,000×g for 10 min at 4°C, a fraction enriched with granule neurons was collected between the 35% and 60% Percoll layers. The isolated neurons were washed once with PBS and subjected to electroporation using the Nucleofector™ system (Amaxa Inc., Gaithersburg, MD). Briefly, 5×10^6^ dissociated neurons were spun down at 138×*g* for 5 min at 4°C, resuspended in 100 µL of Rat Neuron Nucleofector™ solution kept at room temperature, combined with 3 µg of plasmid DNA, transferred into a cuvette and electroporated using program G-13 of the Amaxa system. The electroporated neurons were immediately resuspended in 600 µL of DMEM (Dulbecco's modified essential medium) supplemented with 10% fetal bovine serum, penicillin (100 U/mL), and streptomycin (100 ug/mL) and plated onto 24-well culture plates (BD Falcon, BD Biosciences, Franklin Lakes, New Jersey) or chamber glass slides (Nalge Nunc International, Rochester, NY) coated with 100 µg/mL of poly-D-lysine (PDL; Sigma-Aldrich, St. Louis, MO) and 2 µg/mL of laminin (BD Biosciences, San Jose, CA). Four to 6 hours after electroporation, culture medium was replaced with neurobasal-A medium (Invitrogen, St. Louis, MO) with 200 µM L-glutamine, 2% B27 supplement (Invitrogen, Carlsbad, CA), penicillin (100 U/mL) (Invitrogen, Carlsbad, CA), and streptomycin (100 ug/mL) (Invitrogen, Carlsbad, CA). All cells were cultured at 37°C in an atmosphere containing 5% carbon dioxide. Transfected GFP-positive neurons were imaged using a Zeiss LSM510 META confocal microscope (Carl Zeiss Microimaging, Inc., Thornwood, NY). In order to detect endogenous and transfected levels of *GPR3* , P7 rat cerebellar granule neurons (5×10^6^ granule neurons) were electroporated with 3 µg of pHGCX (Empty) or pHGC-GPR3-RHA (GPR3-RHA). The GPR3-RHA construct, which expresses *mRFP* RNA fused with the recombinant *GPR3* RNA, allows the use of the *mRFP*-specific primers for quantitation of recombinant vs. endogenous *GPR3* expression. Neurons were plated onto PDL/Laminin-coated chamber slides. Twenty-four hours after transfection, total RNA was extracted from the neurons and subjected to quantitative real-time RT-PCR using primers and probes specific for rat *GPR3* and *mRFP*. Untransfected neurons (PBS) were also used as a control. The sequences of PCR primers and VIC-labeled TaqMan probes specific for *mRFP* were5′-ACTACGACGCCGAGGTCAAG-3′, 5′-TGGGAGGTGATGTCCAGCTT-3′, and 5′-VIC-ACATGGCCAAGAAGCCCGTGCA-TAMRA-3′. Ready-made FAM-labeled TaqMan probe and primers specific for rat *β-actin* (Rn00667869_m1) were used as an internal control to normalize the data.

### Immunofluorescence analysis

Cerebellar cells were resuspended in Neurobasal A medium supplemented with B27, penicillin-streptomycin, and L-glutamine (2 mM) and plated on PDL-precoated eight-well chamber slides (BD) at the density of 5.0×10^5^ cells/well. Cells were cultured for 48 hr and then fixed with 4% paraformaldehyde. Permeabilization was performed with 0.1% Triton-X, followed by 10% normal donkey blocking serum.

### Cell proliferation assay

Cell proliferation was evaluated using the bromodeoxyuridine (BrdU) cell proliferation ELISA assay (Roche). Rat cerebellar neurons from P5 or P7 neonates were isolated as described above and transferred to PDL-coated 24-well plates (BD) or eight-well Lab-Tek chamber slides (Nunc, Naperville, IL), at the density of 8.0×10^5^ cells/cm^2^. Six hours after plating, medium was changed to Neurobasal A medium supplemented with B27, penicillin-streptomycin, and L-glutamine (2 mM) (Invitrogen). Stimuli were added at the time of medium replacement, and the cells were cultured for the indicated amount of time (48 hr, unless otherwise noted). Cells were pulsed with 10 uM of BrdU (Roche), 12 hr before the end of the culture period. BrdU-detecting ELISA assay was performed according to manufacture's recommendation. The colorimetric signal of each sample was detected and analyzed using a FLUOstar OPTIMA microplate reader (BMG LABTECH Inc., Durham, NC). For anti-BrdU immunostaining, cells were fixed with 4% paraformaldehyde-PBS for 10 min at room temperature followed by 50% formaldehyde in sodium-citrate buffer at 65°C for 2 hours. Sections were then rinsed in 0.1% Triton X-100 in PBS for 20 min and incubated with Rat anti-BrdU antibody (Oxford Biolab, UK) at 4°C overnight. After washing with PBS, the sections were incubated with Rhodamine labeled secondary antibody (1∶400) (Jackson Immunolab, West Grove, PA) for 1 hour at room temperature. Co-localization with transducted GFP-positive neurons were evaluated using a Zeiss LSM510 META confocal microscope (Carl Zeiss Microimaging, Inc.).

### Real time PCR analysis

Total RNA was isolated from transfected cells using TRIzol reagent (Invitrogen). RNA samples were treated with the TURBO DNA-free™ kit (Ambion) and then reverse transcribed into first-strand cDNA by Superscript II™ reverse transcriptase (Invitrogen) using oligo-dT primers according to the manufacturer's protocols. The sequences of PCR primers and VIC-labeled TaqMan probes specific for mouse *GPR3* genes were selected using Primer Express® software (Applied Biosystems, Foster City, CA): 5′-CTGACCGCGTGGCTCTAGA-3′, 5′-CACTTGGGCTGTGAGACATTTC-3′, and 5′-VIC-TGTTCCAGATGGTCAGGGTCCCACTC-TAMRA-3′ for mouse *GPR3*; 5′-CGCCAACTCTCCTCCTCTCTAC-3′, 5′-CGGGTTGATCATGGAGTTGTAA-3′, and 5′-VIC-CCTACCTTACCCTGCTCCCTGC-TAMRA-3′ for rat *GPR3*. Ready-made FAM-labeled TaqMan probes and primers specific for mouse *β-actin* (Mm00607939_s1, Applied Biosystems) or rat *β-actin* (Rn00667869_m1) were used as internal controls. Real-time PCR analyses were carried out in 96-well optical reaction plates using an ABI PRISM® 7900 HT sequence detection system according to the manufacturer's protocol. After 2-min incubation at 50°C, the samples were denatured by a 10-min incubation at 95°C and subjected to 40 cycles of amplification (95°C for 15 s, 60°C for 1 min). The fluorescence signal from each well was normalized using an internal passive reference. The cycle threshold (C_T_) values obtained with the *GPR3*-specific probes and primers were compared with those of *β-actin*-specific probe and primers using the comparative C_T_ method as described in the user manual (User Bulletin #2 for ABI Prism® 7700).

### Cell cycle analysis by flow cytometry

DNA-indices for cell cycle analysis were assayed by multiparametric flow cytometry using standard methods. Analyses were performed using a Becton Dickinson FACScan flow cytometer (BD Biosciences, San Jose, CA) for the detection of cells stained with propidium iodide (PI) and a 488 nm laser with filter combination for GFP. Single cell suspensions were isolated from culture, fixed in methanol, and stained with PI (100 µg/mL in PBS). Each histogram represents 10,000–100,000 cells for measuring DNA-index and cell cycle. Histogram analysis was performed with the CellQuest software (BD Biosciences) for multiparametric calculations and analyses.

### Statistical analyses

Statistical analysis was performed by one-way ANOVA followed by Fisher's PLSD test. *P*<0.05 was considered statistically significant.

## Results

### Exogenous *GPR3* expression inhibits Shh-induced proliferation of cerebellar granule cell precursors (GCP) and correlates with increased p27/kip

**Figure 1:**
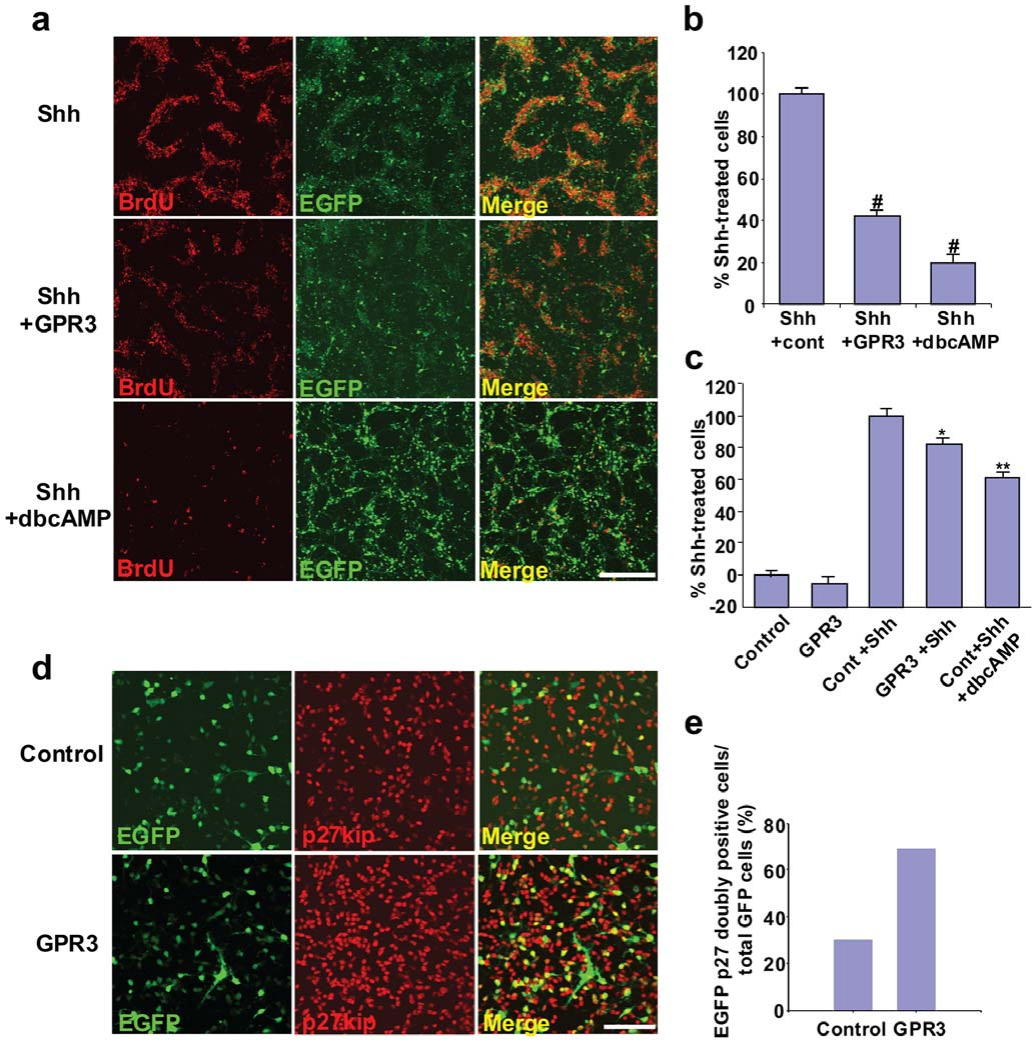
Exogenous *GPR3* partially inhibits Shh-induced proliferation of rodent cerebellar granule neurons and is associated with increased p27/Kip expression. GCPs isolated from rat cerebellum (P7) were electroporated with a *GPR3/EGFP* or control vector using the AMAXA nucleofector system. Shh (1 ug/ml) was then added 6 hours later. Thirty hours later, BrdU (final concentration = 10 µM) was added 12 hours prior to fixation. Cell proliferation was determined using BrdU incorporation assessed by immunohistochemistry with a BrdU-specific monoclonal antibody (panels a, b). dbcAMP (100 µM) was used as a chemical cAMP activator and as a positive control. In panel a, representative fields from dishes are visualized from the Shh and control vector group (top row), Shh and *GPR3/EGFP*-electroporated group (middle row) and the Shh, control vector group and dbcAMP (bottom row). Visualization by BrdU immunohistochemistry (red dye), EGFP fluorescence (green dye), and the combined merged image are shown. Five random fields were selected per each dish (n = 3) from the Shh, Shh+GPR3 and Shh+GPR3+cAMP groups. The number of GFP-positive cells (representing positively transfected neurons) was counted (at least one hundred GFP-positive neurons per each field). Doubly labeled cells (yellow color) were also enumerated per each dish. Values were then expressed as the percentage of doubly-labeled cells out of total GFP positive cells in the Shh+GPR3 group or Shh+GPR3+dbcAMP groups compared to Shh group, i.e. % Shh-treated cells (panel b). In parallel, cell proliferation was also determined using an ELISA assay based on the measurement of BrdU incorporation during DNA synthesis (Panel C) Variations between percentage of cells on the plates in panel b and c are likely due to differences in assays (visual counting vs. counting by colorimetry). For p27/kip1 expression (panel d, e), GCPs isolated from rat cerebellum (P4) were electroporated with the *GPR3/EGFP* expression (bottom row, panel d) or control vector (top row, panel d). p27/kip1 expression was detected by immunohistochemistry, 48 hours later. EGFP and p27/kip1 doubly positive cells were counted in GPR3 and control groups (panel e). *p<0.01;**p<0.001, # p<0.0001, scale bar = 200 µm.

We sought to determine whether *GPR3* expression inhibited proliferative signals (Sonic hedgehog- Shh) and/or correlated with antiproliferative markers (p27/kip), known to be important in postnatal cerebellar development. We electroporated cultured cerebellar granule precursor cells (GCPs), isolated from P7 rat, with a *GPR3/EGFP*-expression vector and then exposed transfected cells to Shh. Although the initial transfection efficiency was likely to be similar for all dishes, GPR3 and cyclic AMP provide a survival advantage to neurons (data not shown and [40]), accounting for the observed increase in GFP-positive cells in those dishes. The proliferation of untransfected control cells was significantly enhanced by exposure to Shh as measured by incorporation of BrdU (fig. 1a). However, exposure to Shh did not stimulate as much proliferation of *GPR3* transfected cells, both visually (Fig. 1a) and upon quantitative enumeration (Fig. 1b). As expected, exposure to high doses of dibutyryl-cyclic AMP abrogated the proliferating action of Shh. This result was also confirmed by using an ELISA assay to detect BrdU-positive cells. As expected, the number of BrdU-positive GCPs was increased by Shh treatment in control, electroporated GCPs (Figure 1c). However, the number of BrdU-positive GCPs was significantly decreased in *GPR3*-transfected GCPs.

We then asked whether exogenous *GPR3* gene expression had an effect on the antiproliferative marker, p27/Kip1. GCPs were electroporated with the *GPR3/EGFP* expression or control vector. Figure 1d shows that, 48 hours after transfection with the *GPR3/EGFP* vector, there was an increase in p27/Kip-expressing cells compared to control that was also confirmed by quantitative evaluation (figure 1e). The mRNA levels of the endogenous *GPR3* and of the transfected recombinant mouse *GPR3* were measured using the rat *GPR3*-specific primers and primers for mouse red fluorescent protein (*mRFP*), respectively. In this experiment, we used the GPR3-RHA construct, which expresses *mRFP* RNA fused with the recombinant *GPR3* RNA, allowing us to use the *mRFP*-specific primers for quantitation of recombinant vs. endogenous *GPR3* expression. The data shows that recombinant *GPR3* is expressed at levels 10-fold higher than those of endogenous *GPR3* (supplemental figure S1). These findings thus showed that exogenous *GPR3* expression into cultured CGPs from postnatal cerebellum partially counteracted the proliferative action of Shh and was associated with expression of the antiproliferative marker (p27/kip).

### *GPR3* mRNA “knock-down” is associated with increased proliferation of CGPs in response to Shh and with reduced expression of p27/Kip

**Figure 2:**
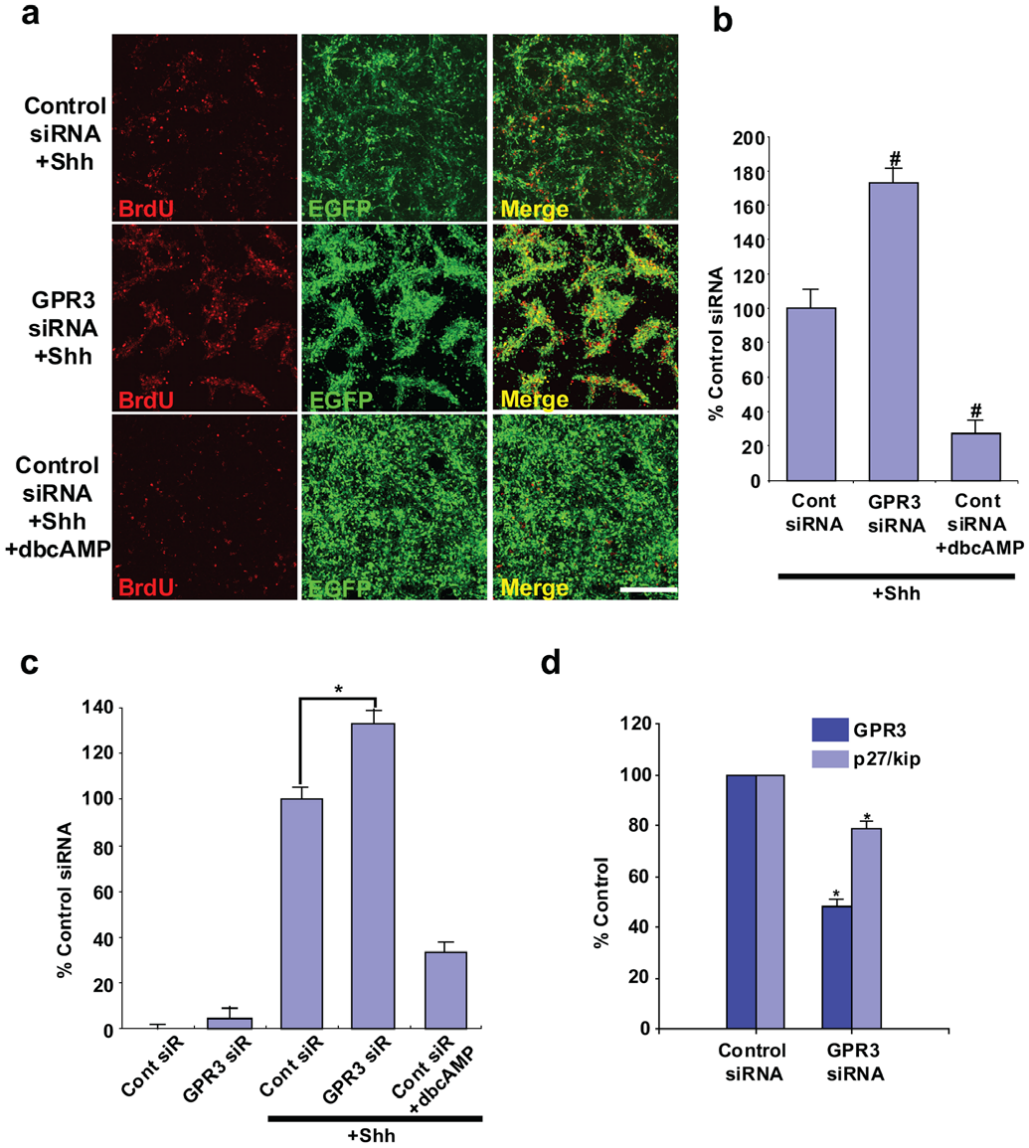
Endogenous GPR3 siRNA-mediated inhibition enhances Shh-induced rodent GCPs proliferation and inhibits p27/kip expression. GCPs were isolated from rat cerebelli (P7) (n = 12–14 to obtain 10^8^ neurons). Cells (5×10^6^) were electroporated with *GPR3* (middle row, panel a) or control siRNA (top row, panel a). To visualize transfected cells, an *EGFP* expression vector was also co-transfected with each siRNA. Shh (1 µg/ml) was administrated 6 hours after. BrdU was added 12 hours prior to fixation. dbcAMP (100 µM) was used as a chemical cAMP activator and as a positive control (bottom row, panel a). In panel a, representative fields from dishes are visualized from the control siRNA+Shh group (top row), *GPR3* siRNA+Shh group (middle row) and *GPR3* siRNA+Shh+dbcAMP (bottom row). Visualization by BrdU immunohistochemistry (red dye), and EGFP fluorescence (green dye) and the combined merged image are shown. Five random fields were selected per each dish (n = 3) from the control siRNA+Shh, Shh+GPR3 siRNA and Shh+GPR3 siRNA+cAMP groups. The number of GFP-positive cells (representing positively transfected neurons) was counted (at least one hundred GFP-positive neurons per each field). Doubly labeled cells (yellow color) were also enumerated per each dish. Values were then expressed as the percentage of doubly-labeled cells out of total GFP positive cells in the Shh+GPR3 group or Shh+GPR3+dbcAMP groups compared to Shh group, i.e. % Shh-treated cells (panel b). In parallel, cell proliferation was also determined using an ELISA assay based on the measurement of BrdU incorporation during DNA synthesis (Panel C). For p27/kip expression, *GPR3* siRNA or control siRNA were electroporated in P4 GCPs (panel d). The mRNA of GPR3 and p27/kip1 was analyzed by real time PCR, 48 hours after transfection and each value was normalized to beta-actin expression. The expression of GPR3 was reduced to approximate 40% of the control siRNA value and this inhibited p27/kip1 mRNA expression by 20%. # p<0.0001; *p<0.001, scale bar = 200 µm

We then asked if inhibition of endogenous GPR3 expression stimulated Shh-induced proliferation of CGPs. We had previously shown that GPR3 was up-regulated postnatally in cerebellar granule neuron precursors [40]. To determine if endogenous *GPR3* expression contributed to Shh-induced GCP proliferation, *GPR3* expression was knocked down by siRNA by a value that was ∼60% that of control (see bar in figure 2d). After addition of Shh, proliferation of GCPs with “knocked-down” *GPR3* was significantly elevated compared to that of control GCPs, both visually (figure 2a) and quantitatively (figure 2b). As expected, the addition of dibutyril cyclic AMP (dbcAMP) in the medium abolished the proliferative action of Shh in *GPR3*-siRNA transfected GCPs. BrdU immunohistochemistry also confirmed these results (Fig. 2c). In addition, downstream effects of Shh signaling, such as Gli1 mRNA expression, were significantly reduced upon siRNA inhibition of *GPR3* (data not shown). To provide further evidence for modulation of antiproliferative effects by GPR3, we also evaluated effects on *p27/kip* gene expression. Figure 2d shows that expression of p27/Kip was reduced in these cultures, with only an approximate 60% “knock-down” of *GRP3* mRNA. Taken in conjunction, this evidence thus suggested a significant role for *GPR3* expression in down-regulating the proliferative effect of Shh and up-regulating p27/Kip gene expression in CGPs.

### GPR3 is expressed during postnatal cerebellar development

**Figure 3:**
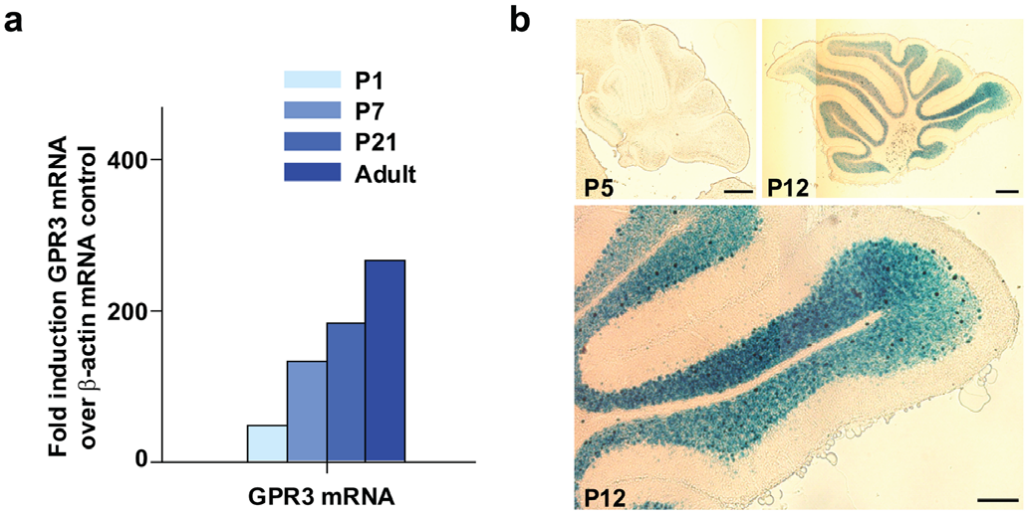
GPR3 expression during postnatal rat and mouse cerebellar development. Panel a: Total mRNA was extracted from rat cerebelli (N = 12–15 per time point) at 4 different developmental stages (P1, P7, P21) and adult (7 to 8 weeks). RNA samples were subjected to quantitative RT-PCR analysis using probes and primers specific to rat *GPR3*. Data were adjusted using *β-actin* mRNA as control. Panel b: To determine the distribution of GPR3 in the developing postnatal cerebellum, a *GPR3−/−; LacZ +/+* mouse was employed, where the *E. coli LacZ* gene was substituted into the GPR3 locus and was thus under transcriptional control of the endogenous GPR3 promoter. Staining for β-galactosidase expression revealed increased activation of transcriptional activity of the GPR3 promoter in the internal granular layer of cerebellum from P5 to P12 stages of postnatal development. Scale bar = 100 µm.

The distribution and expression of mouse *GPR3* in the central nervous system has been previously reported by us[40]. To determine whether GPR3 also plays a role in the developing cerebellum *in vivo*, we first sought to determine if *GPR3* mRNA was expressed postnatally (P1, P7, and P21) in the adult cerebellum. Quantitative RT-PCR showed up-regulation of *GPR3* mRNA in rodent cerebellum from birth until adulthood (Fig. 3A). To determine the pattern and distribution of *GPR3* promoter activity in the developing cerebellum, a genetic model using *GPR3−/−; LacZ+/+* mice was employed. In these mice, the *GPR3* gene is genetically substituted by a *LacZ* gene under control of the endogenous *GPR3* promoter. Figure 3B reveals that *LacZ* gene expression and, thus, *GPR3* gene promoter activity was gradually up-regulated post-natally almost exclusively in the internal granular layer (IGL). A very small number of cells displayed *GPR3* promoter activity in the molecular layer (ML). These results thus indicated that *GPR3* gene expression was up-regulated postnatally and that *GPR3* promoter activity was observed to increase primarily in the IGL after birth.

### Anti-proliferative effect of GPR3 during postnatal cerebellar development

**Figure 4:**
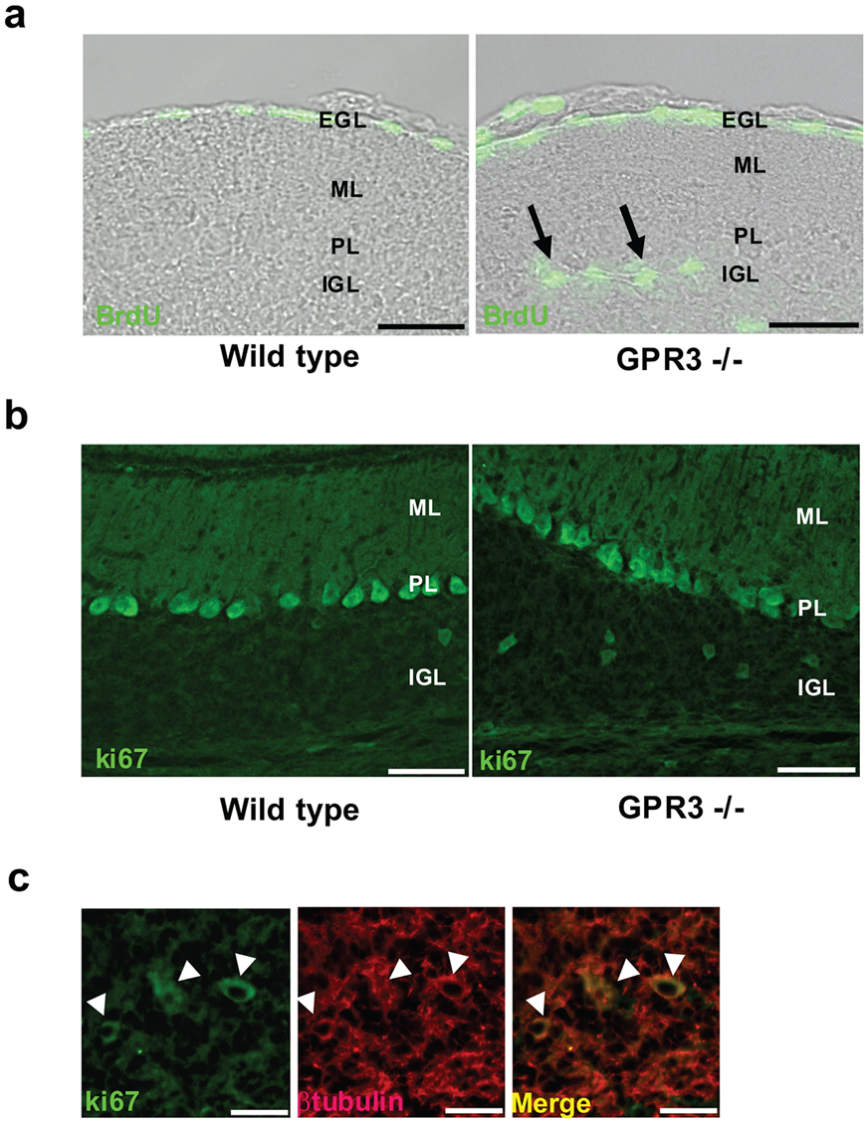
Increased proliferation of granule precursor cells in the IGL of a *GPR3−/−* mouse. In panel a, BrdU was administered to P14 *GPR3−/−* (right panel) or wild type mice (left panel) 4 hours prior to sacrifice. Sections (20 µm) were incubated with a rat anti-BrdU monoclonal antibody and FITC-labelled anti-rat secondary antibody. Fluorescent cells are indicated by arrows in the IGL of *GPR3−/−* mice but not in wild-type mice. As expected, meningeal cells on cerebellar surface also showed BrdU incorporation. In panel b, parallel sections were stained with an anti-ki67 antibody to detect proliferating cells in IGL. Positive Ki67 cells were visualized in IGL of *GPR3−/−* vs. wild-type mice. In panel c, merged images of Ki67 positive cells and βtubulin positive are shown in the right subpanel. White arrowheads point to doubly positive cells. Scale bar = 100 µm (panel a,b) and 20 µm (panel c).

To determine if *GPR3* gene expression was associated with an anti-proliferative effect in the postnatal cerebellum, we utilized the 5-bromo-2′ deoxyuridine (BrdU) pulse chase labeling technique. When BrdU was injected 4 hours prior to the sacrifice of P14 mice, the number of BrdU-positive cells was increased in the IGL of *GPR3*−/− versus wild-type type (Figure 4a). Quantitatively, this increase was significant in all IGL areas of cerebellum except for 10Cb (Table 1). Interestingly, there was no significant difference in the number of BrdU-positive cells in the molecular layer (ML) or white matter (WM) of the cerebellum between wild type and *GPR3*−/− mice. Similar results were also obtained using P12 *GPR3*−/− or wild type mice (data not shown).

**Table 1:** BrdU positive cells per cerebellar lobe.

	2Cb	3Cb	4&5Cb	6&7Cb	8Cb	9Cb	10Cb	Sum
**Wild type**	**4.4±0.91**	**6.5±1.0**	**13.8±1.4**	**15.4±1.9**	**5.2±0.72**	**8.1±0.79**	**3.7±0.59**	**57.0±3.4**
**GPR3 −/−**	**8.2±1.1**	**10.7±1.8** *	**19.3±2.5** *	**25.8±2.0** *	**11.6±1.4** *	**16.7±1.8** **	**3.2±0.56**	**95.4±8.0** *

* p<0.05.

** p<0.001 (Mean±SE).

To provide further confirmation of increased proliferation in *GPR3−/−* mice, we employed immunohistochemical staining for the proliferative markers, Ki67 and phospho-Histone H3. Figures 4B and Table 2 show that the number of Ki67-positive cells was increased in the IGL of GPR3 knockout mice compared with wild type mice at P14. A similar finding was also observed with phospho-Histone H3 immunohistochemistry (data not shown). To determine the lineage of Ki67- positive cells, double immunohistochemical staining was performed using a neuronal (βtubulin) marker. Ki67-positive cells co-localized with βtubulin -positive cells (Fig. 4c). These findings thus confirmed that *GPR3* gene expression was associated with a neuronal antiproliferative effect in the IGL of the postnatal mouse cerebellum.

**Table 2:** Ki67 positive cells per cerebellar lobe.

	2Cb	3Cb	4&5Cb	6&7Cb	8Cb	9Cb	10Cb	Sum
**Wild type**	**7.0±0.55**	**11.4±1.9**	**12.0±1.2**	**5.8±0.58**	**5.8±0.58**	**13.6±2.8**	**3.2±0.97**	**58.8±5.4**
**GPR3 −/−**	**11.8±1.5** *	**21.8±2.5** **	**27.6±2.2** **	**17.8±0.97** **	**8.2±1.7**	**27.4±2.3** **	**7.8±1.5**	**122.4±8.9** **

* p<0.05.

** p<0.001 (Mean±SE).

**Figure 5:**
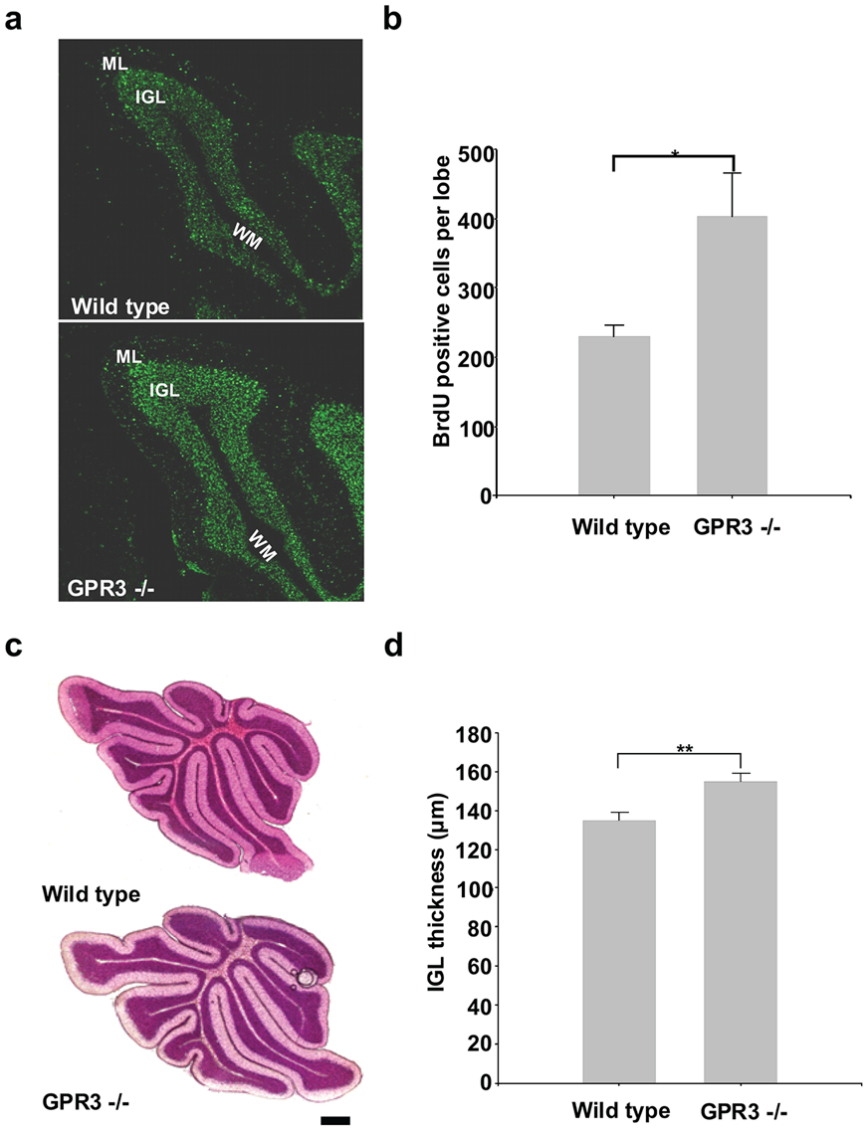
The IGL of GPR3−/− mice is hyperproliferative and enlarged. Wild-type and *GPR3−/−* mice were administered BrdU at P5. Thirteen days later, mice were sacrificed and cerebelli were stained with a BrdU antibody. In panel a, BrdU staining is shown primarily in IGL of wild-type and, even more so, of *GPR3−/−* mice. In panel b, BrdU positive cells in the IGL were enumerated in 9Cb lobe of wild-type vs. *GPR3−/−* mice. In panel c, IGL thickness was visually evaluated in wild-type vs. *GPR3−/−* mice (P14) after hematoxilyn and eosin stain. Thickness of the IGL in 9Cb lobe (P14) was measured in wild-type vs. *GPR3*−/− mice (P14). * = p<0.05; ** = p<0.01. Scale bar = 100 µm.

In order to determine if proliferating neurons in the IGL of *GPR3−/−* mice led to an anatomic difference, BrdU was administered to P5 mice that were then sacrificed 13 days later. At P18, the number of BrdU positive cells in the IGL layer of *GPR3−/−* mice was greater than that in wild type mice (Fig. 5a). This difference was significant upon quantitation (Fig. 5b). The continued neuronal proliferation did result in anatomical thickening of the IGL layer, both visually (Fig. 5c) and quantitatively.(Fig. 5d). These results thus confirmed that the continued neuronal proliferation in the cerebellum of *GPR3−/−* mice did result in anatomical widening of the IGL layer.

### *In vivo* expression of p27/Kip1 is inhibited in *GPR3−/−* mice during postnatal development

**Figure 6:**
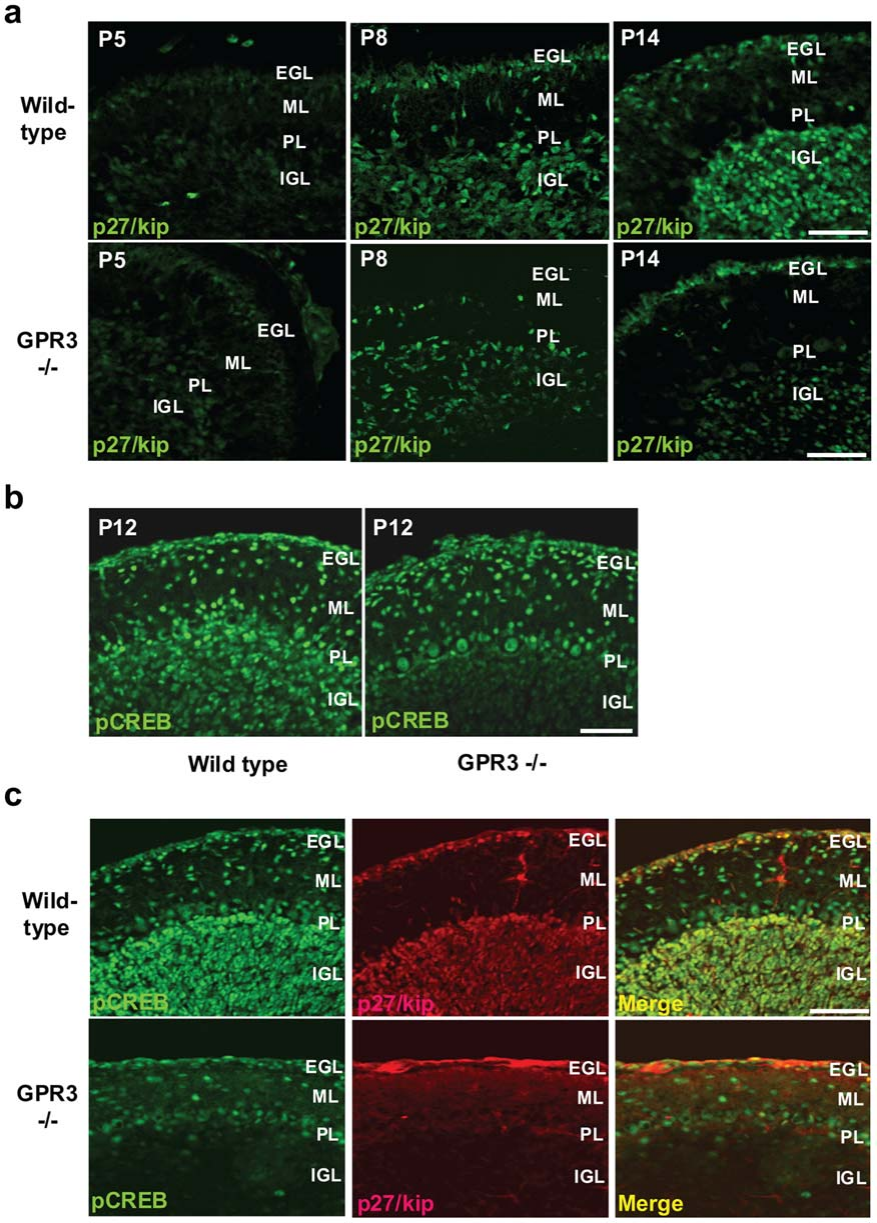
Expression of p27/kip and phosphoCREB expression during postnatal cerebellar development. In panel a, cerebelli from *GPR3−/−* or wild type mice were harvested at P5, P8, and P14. p27/kip1 immunohistochemistry was detected in IGL (as well as other layers) but was visually more readily apparent in wild-type vs. *GPR3−/−* mice at all stages. In panel b, pCREB was detected by immunohistochemistry in wild-type and *GPR3−/−* mice (P12). While pCREB, was detected in the molecular layer (ML) and IGL of widl-type mice, it was not as readily visible in the IGL of the *GPR3−/−*. In panel c, co-localization of pCREB and p27/kip was determined by double immunofluorescence. Scale bar = 50 µm.

To provide additional confirmatory evidence for a regulatory relationship between GPR3 and p27/Kip, we sought to determine the expression of p27/Kip1 in wild type or GPR3−/− mice during postnatal cerebellar development (P5, P8 and P14). The expression of p27/Kip1 in wild-type P5 mice was scarce and observed primarily in IGL in P5 cerebellum (figure 6A). However, this expression visibly increased at the P8 and P14 time points in IGL, but also in the inner side of EGL and in migrating GCPs in ML. In contrast, in *GPR3−/−* mice the expression of p27/Kip1 was visibly reduced in the EGL and IGL of P5, P8 and P14 cerebella compared to that of wild-type mice at the same time points (figure 6a). These data thus indicate that GPR3 gene expression was involved in the regulation of p27/Kip gene expression in the developing postnatal mouse cerebellum.

Since a downstream effect of elevated cAMP in neuronal cells consists of phosporylation of the CREB transcription factor, we also determined whether levels of phosphorylated CREB were decreased in the *GPR3−/−* mice. Figure 6b shows that phospho-CREB expression could be detected in the IGL and ML of P12 wild-type mice. However, this expression was visibly reduced in *GPR3−/−* mice. In addition, p27/Kip and phosphoCREB expression were observed to co-localize in cerebellar granule neurons in the IGL and ML , while there was little or no expression of either in the IGL and ML of *GPR3−/−* mice (figure 6c). Taken in conjunction, this data thus provided evidence for a cAMP-mediated regulatory effect of GPR3 expression on p27/kip expression in the developing cerebellum.

Finally to provide further evidence for effects of GPR3 on cell cycle kinetics, DAOY medulloblastoma cells were transfected with control or *GPR3* expressing vector. The population of cells in G0/G1 phase increased by approximately 10% and that in S phase decreased by an equal amount in *GPR3*-transfected DAOY cells (Table 3). These findings thus showed that GPR3 functioned to promote G1 arrest during the cell cycle.

**Table 3:** Cell cycle analysis for GPR3 expressing DAOY cell.

	G0-G1 phase	S phase	G2-M phase	total
**Control**	52.08%	38.29%	9.63%	100%
**GPR3**	62.07%	25.17%	12.76%	100%
**dbcAMP**	62.42%	24.63%	12.95%	100%

## Discussion

GPR3 is a member of a family of G-protein couple receptors whose activation of PKA and subsequent increase of cyclic AMP level promotes meiotic arrest in the oocyte[38]. Mice deficient in GPR3[38] display premature ovarian aging and loss of fertility[42]. This report also reports another abnormality related to increased proliferation of CGPs in IGL of cerebellum. Our previously published finding that GPR3 was highly expressed in the internal granule layer (IGL) of the rodent postnatal cerebellum led us to hypothesize that GPR3 was one of the factors responsible for inhibition of granule cell precursors' proliferation and induction of their terminal differentiation during development[40]. In this report, we show for the first time that *GPR3* expression: **1)** decreases Shh-induced proliferation and increases p27/Kip expression of GPCs when added exogenously, while it promotes the opposite effect when “knocked- down” by siRNA; **2)** gradually increases postnatally in IGL and, when absent, there is increased proliferation of cells in IGL with a visible neuroanatomical abnormality; and **3)** is linked in vivo to induction of p27/Kip, previously linked to CGP cell cycle exit, and to an increase in phosphorylated CREB, suggesting that its action is mediated by cyclic AMP. Therefore, these results indicate that GPR3 represents one of the molecules responsible for the regulation of the antiproliferative and differentiation program in the postnatal cerebellum.

*In vitro*, increases of GPR3 gene expression by exogenous means did not affect proliferation of postnatal GPCs suggesting that by itself the antiproliferative action of the gene was already at its peak. However, addition of more GPR3 did significantly inhibit the proliferative action of Shh. Although this inhibition seems to be only partial, electroporation experiments may be under-estimating its true magnitude. First of all, the efficiency of electroporation is at best about 50% of cells on the plate, while the effect of Shh, or of the positive control dibutyryl cyclic AMP, is likely to be on all cells on the plate. Second, more detailed dose-response assays could have been performed to determine the stoichiometry of *GPR3* gene expression inhibition of Shh-induced proliferation. In spite of this, the experiment performed still showed significant inhibition of Shh action by GPR3, thus answering the posited question. Third, it is likely that GPR3 is not the only mediator of inhibition of Shh-induced action and that other factors (for instance, other GPRs or PACAP) and signaling pathways are also required to completely abolish GPR3 action. Finally, experiments *in vitro* may not faithfully recapitulate *in vivo* events, due to artifacts of tissue culture. Similar considerations apply to the siRNA experiments. To really determine if GPR3 possesses a significant role in cerebellar development, we did employ studies in wild-type and *GPR3−/−* mice. These studies clearly showed that *GPR3* gene expression expands significantly during the time-course of postnatal development of rats and mice, particularly in the IGL and, when not present, CGPs in IGL are hyper-proliferative. This results in a visible neuro-anatomic difference (enlarged IGL) with decreased expression of p27/kip, as predicted by the *in vitro* studies. Taken in conjunction, thus, these *in vivo* studies do establish GPR3 as a mediator of anti-proliferative signals in CGPs.

We indirectly linked *in vivo* the down-stream effects of GPR3 on Shh and p27/Kip by evaluating phosphorylated CREB in IGL as a marker of cyclic AMP activity. The evidence linking GPR3 expression with cAMP activation in CGPs is indirect. However, GPR3 activation of cAMP has been demonstrated in a variety of cells[37], [43], thus making it likely to also be occurring in CGPs. The findings did show a visually distinct loss of phosphorylated CREB in *GPR3−/−* mice, in agreement with a model where GPR3 activation stimulates cAMP-mediated mechanisms that antagonize Hedgehog signaling and stimulate p27/Kip expression. The interplay of signaling pathways is a critical component in the development of the central nervous system. Cerebellar development has been extensively studied over the decades and the complex interplay of proliferative signals provided by the Hh pathway have revealed a cascade of events regulating the growth of cerebellar neurons, including granule precursors. Interestingly, regulatory signals related to inhibition of proliferation and stimulation of differentiation have not been elucidated, as fully. Adenylate cyclases, such as PACAP and PKA, have been shown to counteract Shh signaling and elevated cyclic AMP levels signal cell cycle exit and terminal differentiation of CGPs[30], [32]. P27/Kip activity also provides a signal for inhibition of proliferation, counteracting Hh[34]. However, no known upstream mediators of PKA and p27/Kip activity have been identified. The findings in this report indicate that GPR3 may represent this upstream mediator. Its genetic absence in mice leads to abnormal proliferation of GPCs postnatally, to an anatomically enlarged IGL, and to a downregulation of p27/Kip levels. When added exogenously GPR3 inhibits Shh-mediated proliferation of these cells and is also associated with p27/Kip upregulation, leading to cell cycle exit. GPR3 belongs to a family of presumably constitutively active G-protein coupled receptors previously linked to oocyte meiotic arrest and to neurite extension in rat neurons[38], [40]. Therefore, the implications of our findings provide a much larger role for this receptor in the development of the CNS. Future studies should elucidate the regulation of its expression in CGPs and the significance of its constitutive activity in these cells with its possible linkage to CGP development and differentiation.

## Supporting Information

Figure S1 Detection of endogenous vs. exogenous GPR3 mRNA from figure 1. In order to detect endogenous and transfected levels of GPR3, P7 rat cerebellar granule neurons (5×106 granule neurons) were electroporated with 3 µg of pHGCX (Empty) or pHGC-GPR3-RHA (GPR3-RHA). The neurons were plated onto PDL/Laminin-coated chamber slides. Twenty-four hours after transfection, total RNA was extracted from the neurons and subjected to quantitative real-time RT-PCR using primers and probes specific for rat GPR3 and mRFP. (panel f)
(0.25 MB TIF)
