# Supplementary figures and images for: The Gs-Linked Receptor GPR3 Inhibits the Proliferation of Cerebellar Granule Cells during Postnatal Development
Source: PLoS One. 2009 Jun 15;4(6):e5922. doi: 10.1371/journal.pone.0005922 (PMC2691605; doi:10.1371/journal.pone.0005922)

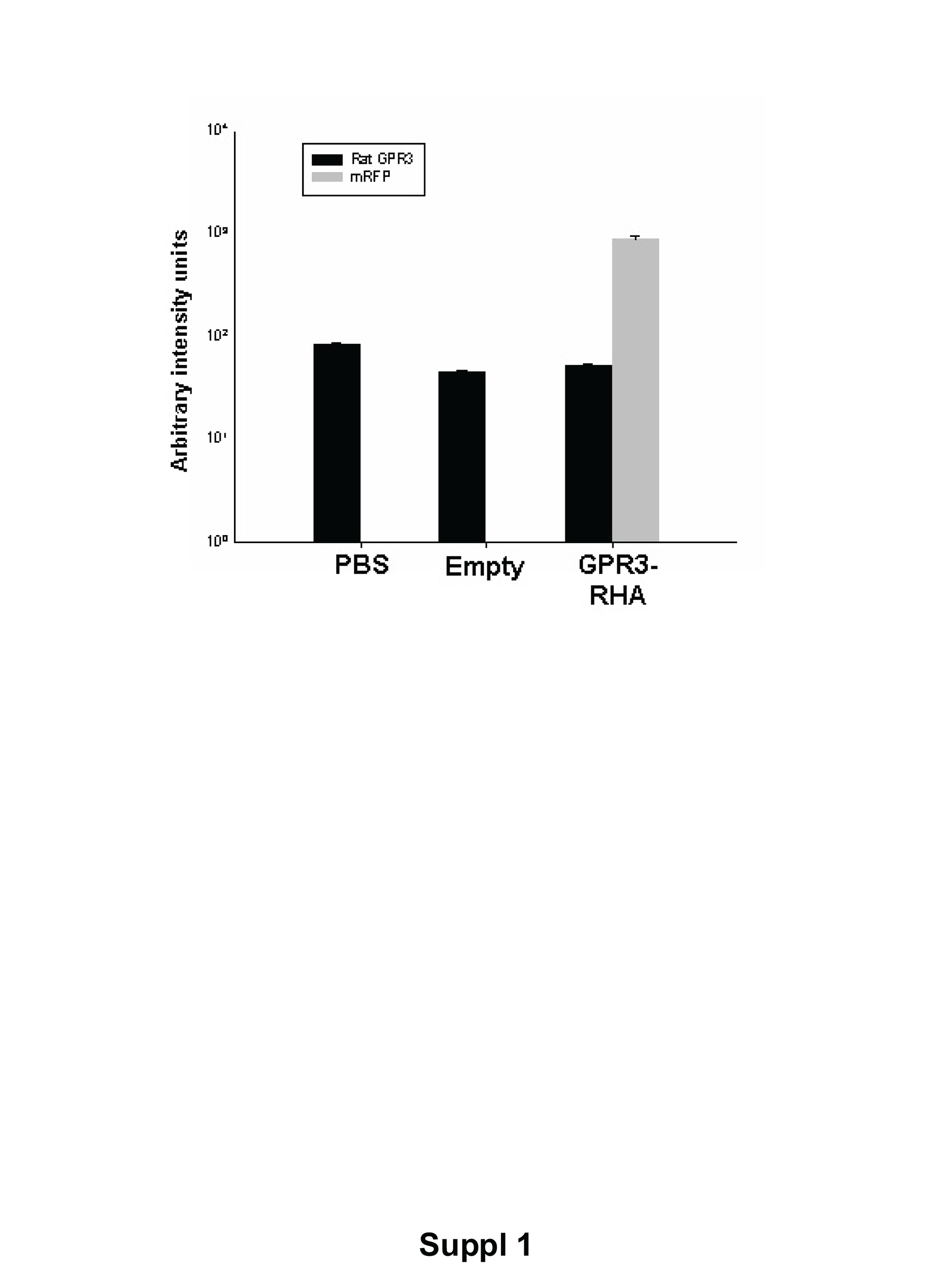

Supplement: Figure S1 — Detection of endogenous vs. exogenous GPR3 mRNA from figure 1. In order to detect endogenous and transfected levels of GPR3, P7 rat cerebellar granule neurons (5×10^6^ granule neurons) were electroporated with 3 µg of pHGCX (Empty) or pHGC-GPR3-RHA (GPR3-RHA). The neurons were plated onto PDL/Laminin-coated chamber slides. Twenty-four hours after transfection, total RNA was extracted from the neurons and subjected to quantitative real-time RT-PCR using primers and probes specific for rat GPR3 and mRFP. (panel f) (0.25 MB TIF) [file pone.0005922.s001.tif]
